# A Recombinant Fusion Construct between Human Serum Albumin and NTPDase CD39 Allows Anti-Inflammatory and Anti-Thrombotic Coating of Medical Devices

**DOI:** 10.3390/pharmaceutics13091504

**Published:** 2021-09-18

**Authors:** Meike-Kristin Abraham, Elena Jost, Jan David Hohmann, Amy Kate Searle, Viktoria Bongcaron, Yuyang Song, Hans Peter Wendel, Karlheinz Peter, Stefanie Krajewski, Xiaowei Wang

**Affiliations:** 1 Clinical Research Laboratory, Department of Thoracic, Cardiac and Vascular Surgery, University Hospital Tübingen, 72076 Tübingen, Germany; meike-kristin.abraham@hotmail.de (M.-K.A.); elenabarb.jost@gmail.com (E.J.); hans-peter.wendel@med.uni-tuebingen.de (H.P.W.); stefanie.krajewski@med.uni-tuebingen.de (S.K.); 2 Atherothrombosis and Vascular Biology, Baker Heart & Diabetes Institute, Melbourne, VIC 3004, Australia; jan_david_hohmann@outlook.com (J.D.H.); asearle@student.unimelb.edu.au (A.K.S.); viktoria.bongcaron@baker.edu.au (V.B.); 3 Molecular Imaging and Theranostics Laboratory, Baker Heart & Diabetes Institute, Melbourne, VIC 3004, Australia; yuyang.song@baker.edu.au; 4 Department of Medicine, Monash University, Melbourne, VIC 3800, Australia; 5 Department of Cardiometabolic Health, University of Melbourne, Melbourne, VIC 3010, Australia; 6 La Trobe Institute for Molecular Science, La Trobe University, Melbourne, VIC 3086, Australia

**Keywords:** albumin, anti-thrombotic, CD39, coating of medical devices, stent coating, therapeutic fusion protein

## Abstract

Medical devices directly exposed to blood are commonly used to treat cardiovascular diseases. However, these devices are associated with inflammatory reactions leading to delayed healing, rejection of foreign material or device-associated thrombus formation. We developed a novel recombinant fusion protein as a new biocompatible coating strategy for medical devices with direct blood contact. We genetically fused human serum albumin (HSA) with ectonucleoside triphosphate diphosphohydrolase-1 (CD39), a promising anti-thrombotic and anti-inflammatory drug candidate. The HSA-CD39 fusion protein is highly functional in degrading ATP and ADP, major pro-inflammatory reagents and platelet agonists. Their enzymatic properties result in the generation of AMP, which is further degraded by CD73 to adenosine, an anti-inflammatory and anti-platelet reagent. HSA-CD39 is functional after lyophilisation, coating and storage of coated materials for up to 8 weeks. HSA-CD39 coating shows promising and stable functionality even after sterilisation and does not hinder endothelialisation of primary human endothelial cells. It shows a high level of haemocompatibility and diminished blood cell adhesion when coated on nitinol stents or polyvinylchloride tubes. In conclusion, we developed a new recombinant fusion protein combining HSA and CD39, and demonstrated that it has potential to reduce thrombotic and inflammatory complications often associated with medical devices directly exposed to blood.

## 1. Introduction

Cardiovascular diseases such as ischemic heart disease and stroke are the world’s leading causes of death. The World Health Organization states that 16% of total deaths can be traced back to these diseases [1]. Treatment of patients with cardiovascular problems often includes the invasive application of medical devices. Often these medical devices will be directly exposed to blood, e.g., vascular grafts, stents, permanently implantable biosensors such as pacemakers and defibrillators. The biomaterials used for blood-contacting devices represent foreign surfaces to human blood and therefore have the potential to induce specific inflammatory and pro-thrombotic reactions that can lead to clinical complications. The underlying pathological mechanisms of these complications are surface-induced reactions of plasma proteins, platelets and leukocytes. Uncoated medical devices often adsorb blood plasma proteins, such as fibrinogen, on their surfaces, thereby inducing an inflammatory process, platelet adhesion and activation of the coagulation [2,3,4,5,6]. Adsorbed proteins further mediate platelet aggregation and, in combination with fibrin, can form a platelet-fibrin thrombus [7]. The activation of platelet aggregation and the coagulation cascade may lead to severe and life-threatening thrombosis on the surfaces of biomaterials [3,7].

Both long-term medical devices (often used for a patient’s lifetime) and short-term blood-contacting systems (mainly used for short-term treatment of critically ill patients) need to be examined for their haemocompatibility and thrombogenicity. During extracorporeal membrane oxygenation, the patient’s blood comes into contact with foreign material such as silicone, polyvinylchloride (PVC) or polypropylene. In a heart–lung machine, the contact of blood with the surfaces of PVC (used for tubing) and polypropylene (used in oxygenators) are the main reasons for postoperative thrombotic and bleeding complications [8,9].

Therefore, major efforts have been undertaken by material scientists and engineers with the aim of designing medical device surfaces that can resist adsorption of blood proteins and adhesion of cells, and thus be less thrombogenic and pro-inflammatory [3]. Different materials and surface coatings have been developed to enhance biocompatibility and reduce device-associated complications [2,3]. Surface modification strategies are classified in two groups: (1) surface passivation; and (2) bioactive surface coatings or treatments [2,10]. With the passivation strategy, physical and chemical modifications are made to the materials and surfaces to reduce their inherent thrombogenicity. Bioactive surface coatings are achieved by permanent immobilization via an active agent or drug to directly inhibit the coagulation cascade and prevent neointimal hyperplasia [11,12,13]. In addition to these, administration of anti-platelet or anti-coagulation therapeutics is used as a treatment to prevent device-induced thrombosis [13,14,15,16].

Other considerations in relation to long-term blood-exposed devices include mechanical adaption to stress. Aortic valve prostheses need to resist constantly changing pressures and high shear stress [17,18]. Permanent implantation of stents for treatment of cardiovascular diseases have shown that drug-eluting stents (DES), with a surface coating of drugs, polymers, growth factors or proteins, promise a superior healing function compared to bare-metal stents [14,17,19,20]. DES have been shown to improve the outcome of revascularization therapy by preventing neointimal hyperplasia and in-stent restenosis (ISR) [21,22,23]. Although the incidence of ISR can be lessened using DES, there is an increased risk of in-stent thrombosis, which requires the application of dual anti-platelet therapy. Overall, this current stent therapy has been associated with adverse bleeding events, hypersensitivity reactions and delayed endothelialisation after implantation [24,25]. Therefore, there is a clinical need for the development of safe and biocompatible surface coatings that eliminate device-induced thrombosis and inflammation.

Human serum albumin (HSA) is widely used in the medical industry for coating of stents and tubings [26,27]. Being a highly abundant protein in the blood, HSA has been shown to have anti-thrombotic properties and corrosion resistance based on its electrostatic and hydrophilic properties [18,26,28]. Additionally, HSA adsorbs easily on surfaces, prevents endothelial apoptosis, provides antioxidant protection and also inhibits platelet activation and aggregation [27,29,30]. Therefore, albumin has played a central role in many drug delivery systems [31]. HSA coating has also been applied to various biomaterials, including titanium (Ti), stainless steel and nanoparticles [26,28,32,33]. For example, dopamine-modified albumin coating showed an attenuated immune and inflammatory response on xenogeneic grafts [34]. Additionally, Oriňaková et al. demonstrated that bovine serum albumin coating changed the corrosion resistance of sintered iron biomaterials [35].

The ectonucleoside triphosphate diphosphohydrolase-1, an NTPDase (CD39), is a promising anti-thrombotic and anti-inflammatory agent [36,37,38,39,40]. Normally expressed on the surface of endothelial cells (ECs), CD39 prevents platelet activation and attachment through hydrolysis of the phosphate residue of ATP and ADP [38,41,42,43]. ATP triggers pro-inflammatory pathways, so the degradation of ATP to ADP by CD39 reduces the pro-inflammatory effect of ATP. ADP is a major player in the platelet-activation cascade [39,44]. Through further hydrolysis of ADP to AMP by CD73, CD39 is responsible for a shift from a pro-inflammatory to an anti-inflammatory environment [39,40,45]. Several studies have confirmed that CD39 activity is substantively reduced in injured or rejected grafts, and that administration of soluble CD39 may be a useful substitute post implantation [41,46].

Here, we have designed, generated and analysed a novel anti-thrombotic and anti-inflammatory recombinant fusion protein consisting of HSA and CD39 as a highly promising bioactive coating for medical devices and PVC tubes to guarantee an active, safe and natural interface between blood and medical devices.

## 2. Materials and Methods

A more detailed description of the methods is provided in the Supplementary Material.

### 2.1. Generation of Recombinant Fusion Construct, Production, Expression of Protein and Purification

Details of HSA-CD39 origin, polymerase chain reaction (PCR)-based fusion, mammalian production (HEK293 cells) and purification are provided as Supplementary Methods. The quantity of the purified protein was measured using a Pierce Protein Assay Kit (ThermoFisher Scientific, Waltham, MA, USA). The samples from purification steps were loaded onto a 12% sodium dodecyl sulfate–polyacrylamide gel for electrophoresis under denaturing conditions and visualized via Coomassie staining. The same samples were also stained on a Western blot (BioRad, Hercules, CA, USA) using an anti-Penta-His antibody (Roche, Basel, Switzerland) coupled with horseradish peroxidase.

### 2.2. Blood Sampling from Healthy Human Volunteers

All blood sampling procedures were approved by the Research and Ethics Unit of the University of Tübingen, Germany (project number 270/2010BO1) and the Ethics Committee of the Alfred Hospital, Melbourne, Australia. Unless otherwise specified, blood was collected by venepuncture from healthy volunteers who provided informed consent and was anticoagulated with citrate. All subjects were free of platelet-affecting drugs for ≥14 days.

### 2.3. Preparation of Platelet-Rich Plasma

Citrated blood from volunteers was centrifuged at 180× *g* for 10 min. Platelet-rich plasma (PRP) was collected and stored at 37 °C. Before usage it was diluted 1:10 with phosphate-buffered saline plus (PBS+; 100 mg/L calcium chloride, 100 mg/L magnesium chloride; ThermoFisher Scientific, Waltham, MA, USA). Blood and PRP were used within the first 6 h after venepuncture.

### 2.4. Flow Cytometry

The efficiency and functionality of the HSA-CD39 protein were determined using flow cytometry. The protein was incubated with 20 µM ADP (MoeLab, Langenfeld, Germany) or 5 µL PBS for 20 min. The active protein will hydrolyse ADP to AMP. Diluted PRP was added and incubated for 5 min. Platelet activation status was measured by a fluorescein isothiocyanate (FITC)-labelled monoclonal antibody PAC-1 (BD Bioscience, Franklin Lakes, NJ, USA), a R-phycoerythrin (PE)-labelled monoclonal antibody directed against CD62P (P-Selectin) (BD Bioscience, Franklin Lakes, NJ, USA) or their respective isotype antibody controls (ThermoFisher Scientific, Waltham, MA, USA). Samples were fixed using Cellfix (BD Bioscience, Franklin Lakes, NJ, USA) and analysed via fluorescence-activated cell sorting (FACS) Calibur (BD Bioscience, Franklin Lakes, NJ, USA). A total of 10,000 events were acquired in each sample.

### 2.5. ADP Bioluminescence Assay

HSA-CD39′s function to directly hydrolyse ADP was measured using an ATP bioluminescence assay according to the manufacturer’s description (Kit CLS II, Roche, Basel, Switzerland) [39,41]. HSA-CD39 was incubated with 20 μM ADP for 20 min. The remaining ADP was converted to ATP by the pyruvate kinase reaction, and measured using the ATP bioluminescence assay via a microplate luminometer (Mithras LB 940, Berthold Technologies, Bad Wildbad, Germany). Different concentrations of ADP, PBS and HSA (Alburex Human albumin 5%, CSL Behring, Hattersheim am Main, Germany) were also used as controls.

### 2.6. Lyophilisation of Protein

To analyse the possibility of lyophilising the HSA-CD39 protein, different concentrations were lyophilised using the CoolSafe ScanVac (LaboGene ApS, Lynge, Denmark) according to the manufacturer’s description. The lyophilised samples were stored for 14 days at room temperature (RT) before rehydration and analysis of platelet activation using flow cytometry.

### 2.7. Coating of Stent Material for In Vitro Analysis

HSA-CD39 and HSA (CSL Behring, Hattersheim am Main, Germany) proteins were passively adsorbed by the different materials. The samples were diluted in PBS, added, incubated and dried on the materials with HSA-CD39 and HSA (CSL Behring, Hattersheim am Main, Germany). Materials were then stored for 24 h before flow cytometric analysis of CD39 activity. Polystyrene (BD Bioscience, Franklin Lakes, NJ, USA), 316L stainless-steel plates, Ti plates (Acandis, Pforzheim, Germany), polyurethane-coated stents (Acandis, Pforzheim, Germany) and nitinol BlueOxide stents (Acandis, Pforzheim, Germany) were coated with different protein concentrations (0.05 µg, 0.1 µg, 0.25 µg and 0.5 µg) and PBS as the control. Coated 316L stainless-steel plates were washed 3× with PBS and dried again prior to functional testing. Coated Ti plates were sterilized with ethylene oxide (EO) according to the sterilization protocol for medical devices of the University Hospital of Tübingen, Germany. Long-term-coated material was stored at RT. DERIVO nitinol BlueOxide stents (3.3 × 15 mm, Acandis, Pforzheim, Germany) were coated by dip-coating of stents with 100 µg/mL HSA-CD39 in PBS 10× and dried with argon gas between dipping steps. Coated stents were also sterilized by EO according to the sterilization protocol.

### 2.8. Endothelialisation Analysis of Protein-Coated Nitinol BlueOxide Plates

The endothelialisation efficiency of HSA-CD39-coated plates was analysed using nitinol BlueOxide plates coated with 4.0 µg/cm^2^ HSA-CD39. Coated plates were dried at RT followed by sterilisation under UV light for 30 min. Human ECs (hECs) were isolated from saphenous vein biopsies of patients who had undergone coronary artery bypass graft surgery as previously described by Avci-Adali et al. [47]. hECs were cultivated in cell-culture flasks pre-coated with 0.1% gelatine in Vasculife^®^ EnGS EC culture medium (CellSystems, Troisdorf, Germany) containing VascuLife EnGS LifeFactors Kit, 50 mg/mL gentamicin and 0.05 mg/mL amphotericin B (GE Healthcare, Boston, MA, USA). Cells were kept at 37 °C/5% CO_2_ and passaged using trypsin/ethylenediaminetetraacetic acid (EDTA) (0.04%/0.03%, PromoCell, Heidelberg, Germany). For endothelialisation analysis, 150,000 cells/well were seeded on coated nitinol BlueOxide plates and incubated for 48 h. Cells were fixed and stained with 4′,6-diamidino-2-phenylindole (DAPI) (Sigma-Aldrich, Sankt Gallen, Switzerland) and analysed via epifluorescence microscopy (Blue UV2A Nikon Optiphot 2, Tokyo, Japan).

### 2.9. In Vitro Haemocompatibility Testing Using Roller Pump and Modified Chandler Loop Model

To investigate the influence of HSA-CD39-coated nitinol BlueOxide stents (nitinol BlueOxide DERIVO embolisation device, Acandis, Pforzheim, Germany) in vitro, coated plates were loaded into heparin-coated Tygon tubes (Saint Gobain Performance Plastics, Wertheim, Germany). PVC tubes (inner diameter 3.2 mm, length 75 cm) were coated with heparin by Ension (Pittsburgh, PA, USA). Through this model, the haemocompatibility of the coated stents, i.e., activation of the coagulation cascade, the complement system and inflammation, were analysed after perfusion of blood, as described in detail by Krajewski et al. [48]. Human whole blood was anticoagulated with heparin (1.5 IE/mL, Ratiopharm GmbH, Ulm, Germany). Then, each tube was filled with 6 mL freshly heparinised human blood, connected by a silicon connection tubing and circulated by a roller pump (BVP Ismatec, Wertheim, Germany) in a water bath at 37 °C for 60 min at 150 mL/min. For each of the 5 donors, 6 mL heparinised blood was used for baseline measurements before circulation. Before and after circulation, blood was taken, measured with a haematolyser (ABX Micros 60, Axon Lab AG, Baden, Switzerland) for blood cell count and further used for enzyme-linked immunosorbent assays (ELISA) (Echelon Biosciences, Salt Lake City, UT, USA) [45,46]. For measuring thrombin–antithrombin III complex (TAT complex; Enzygnost TAT Micro, Siemens Healthcare, Erlangen, Germany) via ELISA, blood was directly filled in citrate S-Monovettes^®^ (Sarstedt AG & Co, Nümbrecht, Germany) and centrifuged at 1800× *g* at 22 °C for 18 min. Resulting plasma was deep-frozen in liquid nitrogen and stored at −20 °C until performance of ELISA, according to the manufacturer’s description. Stents were prepared for scanning electron microscopy (SEM).

In a second experimental setup, the previously established modified chandler loop was used to test the haemocompatibility of coated ECC tubes [10,48]. PCV tubes (lengths of 50 cm; Raumedic^®^ ECC BloodLine 1/4 × 1/16, Raumedic AG, Helmbrechts, Germany) were coated via rotating incubation with 12 mL of 20 μg/mL (240 μg) HSA or 20 μg/mL (240 μg) HSA-CD39 at RT for 3 h followed by storage at 4 °C overnight. The pH of the protein solutions were adjusted to 4.6 prior to incubation. After incubation, tubes were rinsed with PBS prior to being filled with blood. An untreated tube without blood contact, an untreated tube and a commercially available heparin-coated tube (Carmeda BioActive Surface^®^, Medtronic, Dublin, Ireland) were used as controls. Coated tubes were filled with fresh, pooled and heparinised blood (1 IE/mL) and closed into a ring. Blood was circulated in a water bath at 37 °C for 90 min (30 rotations/min). Afterwards, tubes were washed with PBS and fixed with 2% glutaraldehyde (GA), then PVC pieces were prepared for SEM.

### 2.10. Statistical Analysis

Unless otherwise specified, data are represented as mean ± standard deviation (SD). All analyses containing more than two groups were analysed with one-way analysis of variance (ANOVA), comparing all groups with one another, corrected by post hoc Bonferroni analysis or Dunnett’s/Sidak’s test, and the corrected *p*-values are given. Multiple comparisons were analysed with two-way ANOVA and Dunnett’s multiple comparison. All analyses for two groups were performed using Student’s t-tests. The statistical analyses were performed with the statistical software package GraphPad Prism (version 6.0, GraphPad Software, San Diego, CA, USA). Statistical significance was defined as *p* < 0.05.

## 3. Results

### 3.1. Generation, Production and Enzymatic Activity of Recombinant Fusion Protein HSA-CD39

**Figure 1:**
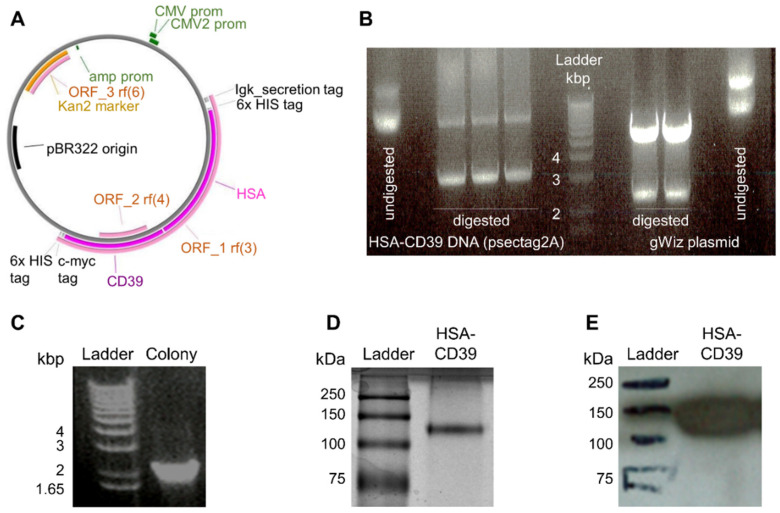
Vector map, generation, purification and characterisation of the HSA-CD39 construct. (**A**) Gene map of the HSA-CD39 construct (8585 bp) within the gWiz vector. The restriction enzymes for inserting the construct are EcoR1-HF and PsPOMI. (**B**) HSA-CD39 cut from pSectag2A (3235 bp) and ligated into gWiz by 1% agarose electrophoresis gel, double digested using EcoR1-HF and PsPOMI. (**C**) Control PCR on 1% agarose electrophoresis gel using gWiz forward primer and Not-1 reverse primer to detect HSA in gWiz (2149 bp). (**D**) Visualized via Coomassie staining, 12% sodium dodecyl sulfate-polyacrylamide gel electrophoresis of the HSA-CD39 construct. (**E**) Western blot analysis using a horseradish-peroxidase-coupled anti-6x-his to detect the 6x-his-tag of the HSA-CD39 construct (141 kDa).

For the generation of our recombinant fusion protein consisting of HSA and CD39, the DNA sequence of HSA was inserted into a previously described pSectag2A vector containing the CD39 sequence [39]. The resulting HSA-CD39 was further digested, purified and inserted into a gWiz vector to yield a higher production rate (Figure 1A). Following double digestion of both constructs, the HSA-CD39 insert was visualised via agarose gel at 3235 bp (Figure 1A). Confirmation of successful molecular biology was made by colony screening of clones via PCR sequencing, where positive clones resulted in a 2149 bp DNA strain (Figure 1C). After DNA sequencing confirmation, the DNA was produced by HEK293F cells and purified afterwards. The protein purity of the HSA-CD39 fusion protein was analysed on SDS–PAGE and a band was observed between the 100 kDa and 150 kDa marks (Figure 1D). Specificity of the HSA-CD39 construct was shown by Western blotting via the use of an anti-Penta-His antibody, which was coupled with horseradish peroxidase (141 kDa, Figure 1E).

**Figure 2:**
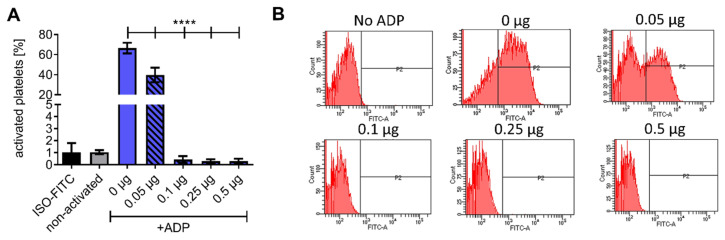
Functionality analysis of different concentrations of the HSA-CD39 construct. (**A**) Functionality analysis using flow cytometry detecting activated platelets through binding of the PAC-1 antibody, showing hydrolysing effects of CD39. (**B**) Representative fluorescence histograms of functionality analysis using flow cytometry. The different groups were compared using repeated-measures ANOVA and Bonferroni post hoc test. Values of 5 independent experiments are shown (% activated platelets ± SD, **** *p* < 0.0001).

The enzymatic activity of the HSA-CD39 fusion protein in hydrolysing ADP to AMP was determined using flow cytometry. While ADP is a platelet agonist, the resulting AMP is unable to activate platelets in vitro. Flow cytometry demonstrated that PAC-1 FITC and anti-CD62P PE bound to 20 µM ADP-activated platelets, but not to non-activated platelets incubated with PBS as control (Figure 2A and Figure S1). By pre-incubating 0.05 µg of the HSA-CD39 protein with 20 µM ADP, we observed a strong reduction in PAC-1 binding as compared to using no protein control (0 µg), indicating successful hydrolysis of ADP to AMP (39.70 ± 7.25 vs. 66.56 ± 5.27, respectively; % activated platelets ± SD, *p* < 0.0001). Higher concentrations of HSA-CD39 (0.1 µg, 0.25 µg and 0.5 µg) resulted in complete dephosphorylation of ADP and therefore showed no PAC-1 binding (0.44 ± 0.28; 0.30 ± 0.16; 0.30 ± 0.20, respectively; % activated platelets ± SD, *p* < 0.0001) (Figure 2A,B).

### 3.2. In Vitro Analysis of Environmental Stability of HSA-CD39 Fusion Protein and Coating

**Figure 3:**
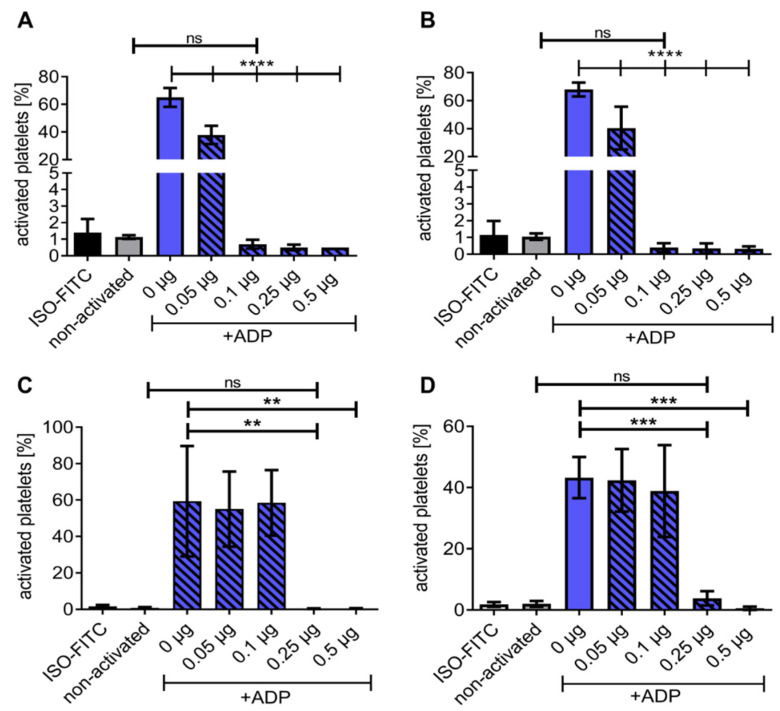
Flow cytometry demonstrating HSA-CD39 functionality when dried in polystyrene tubes and stored at RT for at least 8 weeks. Functionality of HSA-CD39 in hydrolysing ADP is still seen after drying in polystyrene tubes and storage at (**A**) 4 °C for 24 h or (**B**) at RT for 24 h. Flow cytometry was performed to determine the % of activated platelets. (**C**) HSA-CD39 is still functional after 7 days of storage at RT. (**D**) HSA-CD39 is still functional after 8 weeks of storage at RT. The different groups were compared using repeated-measures ANOVA and Bonferroni post hoc tests. ns = non-significant. Values of at least 3 independent experiments are depicted (% activated platelets ± SD, ** *p* < 0.01, *** *p* < 0.001, **** *p* < 0.0001).

HSA-CD39 was dried or lyophilised and stored to determine the ease of storage and handling. Flow cytometry analysis confirmed that our HSA-CD39 protein had active enzymatic properties after being dried in polystyrene tubes (Figure 3A,B). Twenty-four hours after air-drying and storage at RT, rehydration of the HSA-CD39 protein hydrolysed ADP at 0.05 µg, 0.1 µg, 0.25 µg and 0.5 µg, thereby preventing the binding of PAC-1, as compared to samples without HSA-CD39 (40.40 ± 15.28; 0.40 ± 0.26; 0.35 ± 0.30; 0.33 ± 0.15 vs. 67.93 ± 4.96, respectively; % activated platelets ± SD, *p* < 0.0001). Similarly, storage at 4 °C resulted in successful hydrolysis of ADP; therefore, HSA-CD39 at 0.05 µg, 0.1 µg, 0.25 µg and 0.5 µg prevented PAC-1 binding shown by flow cytometry, compared to samples without HSA-CD39 (37.38 ± 6.70; 0.70 ± 0.26; 0.50 ± 0.17; 0.50 ± 0.00 vs. 65.03 ± 6.81, respectively; % activated platelets ± SD, *p* < 0.0001).

The functionality of HSA-CD39 was investigated every week for two months (dried in polystyrene tubes and stored at RT) via flow cytometry (Figure 3C and Figure S2). HSA-CD39 hydrolysed ADP and stopped platelet activation at ≥0.25 µg protein after rehydration, compared to samples without HAS-CD39 after week 1 (0.40 ± 0.17 vs. 59.33 ± 30.28; *p* < 0.001) and week 8 (3.80 ± 2.33 vs. 43.23 ± 6.73; % activated platelets ± SD, *p* < 0.001). The enzymatic properties of HAS-CD39 were similarly active through the 8 weeks of storage.

**Figure 4:**
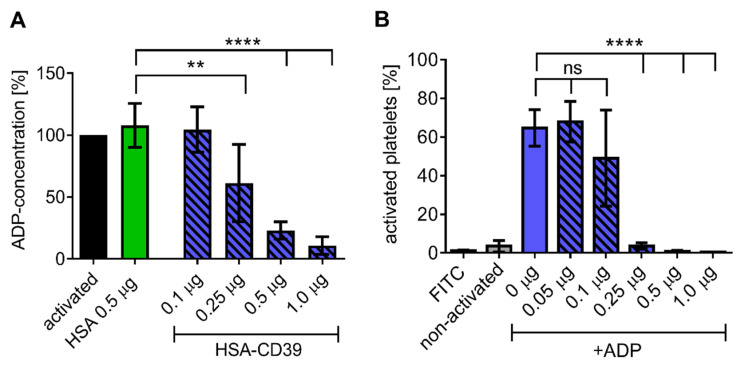
HSA-CD39 shows efficient ADP hydrolysis and can be lyophilised at higher concentrations without reduction in functionality. (**A**) Bioluminescence assay showing a reduced ADP concentration (%) for increased HSA-CD39 concentrations compared to the HSA control (** *p* < 0.01; **** *p* < 0.0001 compared to 0.5 μg HSA control, *n* = 4). Different groups were compared using repeated-measures ANOVA and Sidak’s test. (**B**) Functionality of lyophilised HSA-CD39 shows that high amounts of the fusion protein (>1.0 µg) are still active after lyophilisation (*n* = 4, **** *p* < 0.0001 compared to PBS control). Values of at least 3 independent experiments are depicted.

Direct analysis of ADP dephosphorylation by HSA-CD39 was conducted using an ATP bioluminescence assay. Increasing concentrations of HSA-CD39 resulted in linear and significant reductions in ADP concentration in comparison to the HSA control (Figure 4A). Similar effects were observed using lyophilised HSA-CD39, which demonstrates its long-term stability (Figure 4B).

### 3.3. HSA-CD39 Fusion Protein as an Anti-Thrombotic Coating for Different Medical Materials

**Figure 5:**
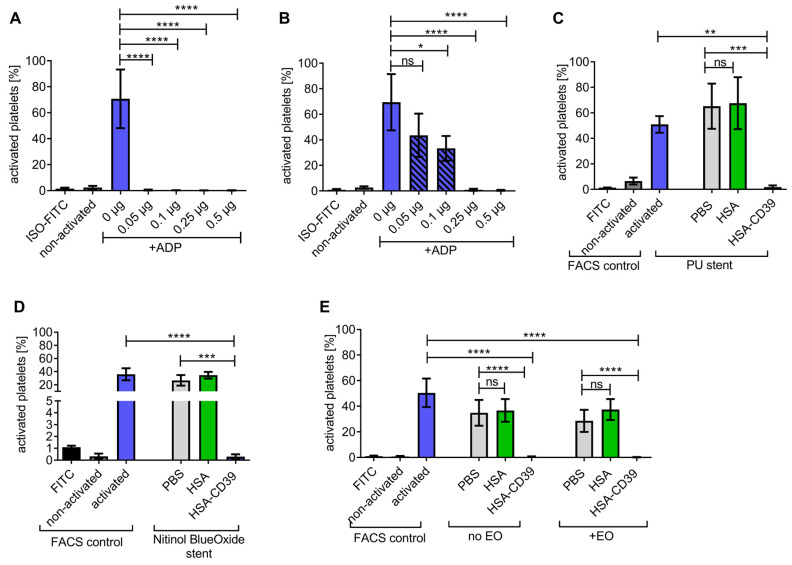
HSA-CD39 can be coated onto stainless steel, polyurethane stents and nitinol BlueOxide stents even after sterilisation with EO without reducing its functionality. (**A**) Significant reductions in platelet activation can be seen with different concentrations of HSA-CD39-coated stainless-steel plates without washing. (**B**) HSA-CD39 coated onto stainless-steel plates displays a reduced but still significant reduction in platelet activation after washing with PBS before analysis in flow cytometry. Bar graphs depict % of activated platelets. (**C**) Dried and coated HSA-CD39 on polyurethane stents as well as nitinol BlueOxide stents. (**D**) Shows effective prevention of platelet activation as analysed via flow cytometry. (**E**) HSA-CD39 and HSA for comparison, coated on Ti plates, shown to be still functional in hydrolysing ADP after EO sterilisation. The different groups were compared using repeated-measures ANOVA and Bonferroni post hoc tests. ns = non-significant. Values of at least 3 independent experiments are depicted (% activated platelets ± SD, * *p* < 0.05, ** *p* < 0.01, *** *p* < 0.001 **** *p* < 0.0001).

HSA-CD39 was coated onto surface materials of medical devices such as stainless steel, polyurethane, nitinol BlueOxide and Ti. Stainless-steel plates were coated at 0.05 µg/mm^2^ (0.25 µg of HSA-CD39 on about 0.5 mm^2^) and resulted in a significant reduction in platelet activation. Direct HSA-CD39 coating on the plates resulted in successful ADP dephosphorylation as compared to non-coated plates (Figure 5A; 0.17 ± 0.06 vs. 70.70 ± 22.55; % activated platelets ± SD, *p* < 0.0001) and similar results were observed when the plates were washed thrice prior to exposure of ADP (Figure 5B; 0.93 ± 0.80 vs. 69.43 ± 21.99; *p* < 0.0001). Likewise, HSA-CD39 coating demonstrated good enzymatic activity for hydrolysing ADP on polyurethane, compared to HSA-coated stents (Figure 5C; 1.88 ± 1.25 vs. 65.23 ± 17.71; *p* < 0.001) and nitinol BlueOxide stents (Figure 5D; 0.30 ± 0.20 vs. 34.53 ± 5.13; *p* < 0.001). Furthermore, we investigated the stability of HSA-CD39 after coating on Ti plates and sterilisation with EO (Figure 5E). HSA-CD39 coating at 0.1 µg/mm^2^ (0.5 µg of HSA-CD39 on about 0.5 mm^2^ of plate) resulted in successful ADP dephosphorylation as compared to the control HSA coating, both before (0.57 ± 0.29 vs. 36.73 ± 8.88; *p* < 0.001) and after EO sterilisation (0.22 ± 0.17 vs. 37.44 ± 8.17; *p* < 0.001). No difference was noted in the function of HSA-CD39 after sterilisation with EO (0.57 ± 0.29 vs. 0.22 ± 0.17; ns).

### 3.4. HSA-CD39 Fusion Protein Coating Allows for Endothelialisation

**Figure 6:**
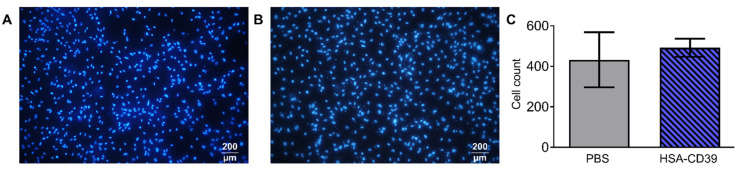
HSA-CD39 coated onto nitinol BlueOxide plates has no negative impact on endothelialisation. Incubation of HSA-CD39-coated nitinol BlueOxide stents with hECs. Analysis performed after 48 h with DAPI staining under epifluorescence microscopy. (**A**) Blank plate incubated with hECs. (**B**) HSA-CD39-coated plate incubated with hECs. (**C**) Quantitative analysis of coated (HSA-CD39) and uncoated (PBS control) plates. Original magnification: 10×. Values of at least 3 independent experiments are depicted.

Fluorescence microscopy images of the HSA-CD39-coated nitinol BlueOxide plates displayed good endothelialisation performance. Sterilised plates were coated with HSA-CD39 or just PBS. hECs were seeded onto the coated plates, followed by incubation for 48 h. No differences between the DAPI-stained hECs regarding cell morphology, cell growth and cell count could be detected compared to the non-coated bare nitinol BlueOxide plates (Figure 6A,B). Additional quantitative analysis of the microscope pictures using ImageJ confirmed this result (Figure 6C).

### 3.5. Haemocompatibility and In Vitro Proof of Function of HSA-CD39-Coated Nitinol Blue Oxide Stents and PVC Tubes

**Figure 7:**
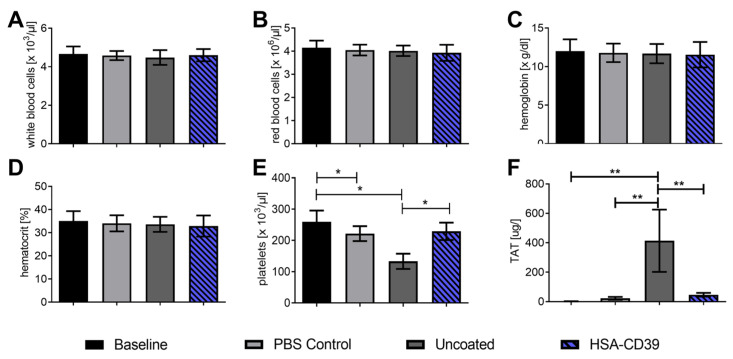
HSA-CD39 coated onto nitinol BlueOxide stents shows no effect on blood haematology or haemocompatibility using a dynamic in vitro thrombogenicity model. Haematology analysis of coated stents before and after circulation for 60 min at 150 mL/min (thrombogenicity model). (**A**) White blood cells. (**B**) Red blood cells. (**C**) Haemoglobin. (**D**) Haematocrit. (**E**) Platelets. (**F**) TAT complex using ELISA measurements. Baseline: directly after venepuncture. Control: tube only. Uncoated: bare nitinol BlueOxide stent. The different groups were compared using repeated-measures ANOVA and Bonferroni post hoc tests (% activated platelets ± SD, * *p* < 0.05, ** *p* < 0.01). Values of at least 4 independent experiments are depicted.

HSA-CD39-coated nitinol BlueOxide stents and uncoated stents were loaded into PCV tubes and incubated with fresh human blood to determine their haemocompatibility and thrombogenicity [22,38]. PVC tubes without stents were used as an additional control. A baseline reading was analysed before the blood was placed into circulation through the stents or tubes. At the endpoint, the blood was collected for comparison analysis. No significant changes were measured in white blood cells, red blood cells, haemoglobin or haematocrit compared to the baseline, the control tube without stent and the uncoated stent (Figure 7A–D). Significant reductions in the number of platelets were found when blood was circulated in the PVC control tube and the uncoated groups, compared to the baseline (221,000.6 ± 23,000.84 and 133,000.2 ± 24,000.3 vs. 259,000 ± 36,000, respectively; number of platelets/µL ± SD, *p* < 0.05). However, no difference was observed in the HSA-CD39-coated nitinol BlueOxide stents as compared to the baseline reading (229,000.0 ± 27,000.74 vs. 259,000 ± 36,000; ns) (Figure 7E). These results indicate that the platelets in the PVC tube control and the uncoated groups aggregated, whereas no aggregation occurred in the HSA-CD39-coated nitinol BlueOxide stents. Activation of the coagulation cascade was determined by measuring the formation of the TAT complex before and after perfusion (Figure 7F). An increased readout of the TAT complex for the uncoated stent group was shown compared to the baseline, the control and also the HSA-CD39-coated stent group (446.4 ± 225.5 vs. 2.37 ± 0.45; 24.02 ± 11.45; 42.72 ± 11.26, respectively; µg/L TAT complex formation ± SD, *p* < 0.01).

**Figure 8:**
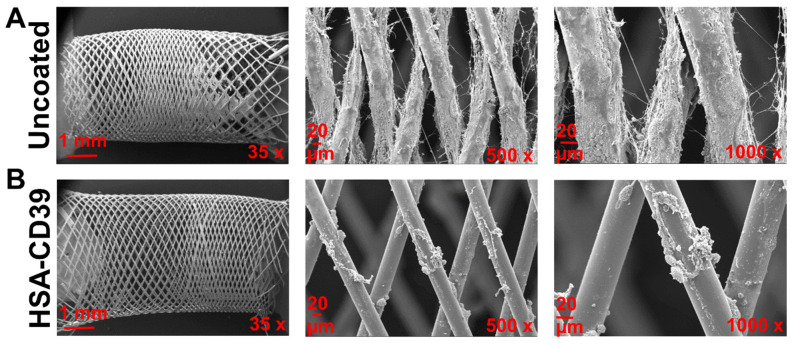
HSA-CD39 coated onto nitinol BlueOxide stents reduces blood cell adhesion during incubation in a dynamic in vitro model. SEM analysis of coated nitinol BlueOxide stents after circulation with human whole blood for 60 min at 150 mL/min (thrombogenicity model). Uncoated: bare nitinol BlueOxide stent. Different magnifications (35×, 500× and 1000×) show the amount of blood cell adhesion.

**Figure 9:**
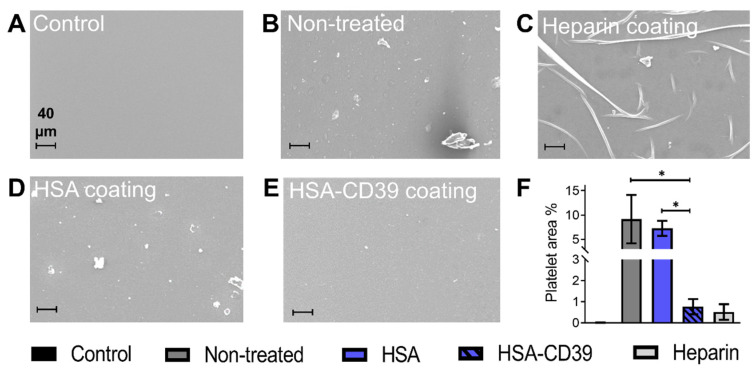
HSA-CD39 coated onto PCV tubes reduces blood cell adhesion as analysed in a modified chandler loop model. SEM analysis of PVC tubes to visualise the adhesion of platelets after circulation with human whole blood. (**A**) PVC tube without blood contact. (**B**) Non-treated PVC tube incubated with blood. (**C**) Commercially available heparin coating (Carmeda BioActive Surface^®^, Medtronic, Ireland). (**D**) HSA-coated PVC tube. (**E**) HSA-CD39-coated PVC tube (magnification: 250×). Less adhesion of platelets was measured in HSA-CD39-coated tubes as compared to HSA or non-treated controls. (**F**). Quantitative analysis of the percentage of area covered by platelets was performed using ImageJ (% activated platelets ± SD, * *p* < 0.05). Values of at least 3 independent experiments are depicted.

After circulation, uncoated and HSA-CD39-coated stents were also analysed via SEM. Representative SEM images of each stent from the same blood donor, displayed at different magnifications, showed distinct differences in the blood cell adhesion (Figure 8). The uncoated stent showed homogenous adhesion of several blood cells, especially platelets, and an increased fibrin network for all blood donors, such that only a few platelets could be detected on the surface of the HSA-CD39-coated stent (Figure 8). SEM imaging of HSA-CD39 coating on the PVC tubes showed a reduction in cell adhesion on the inner surface of the HSA-CD39-coated tube after circulation as compared to the other control groups (Figure 9). In particular, the non-treated and HSA-coated tubes showed more cell adhesion compared to the HSA-CD39-coated tube.

## 4. Discussion

Medical devices that are directly exposed to blood are often associated with inflammation and thrombus formation [2,3]. The lack of biocompatibility of foreign materials triggers inflammatory processes and activation of the coagulation cascade, as well as activation and aggregation of platelets in the blood [49,50,51]. The use of drug-eluting materials and extensive anti-platelet therapy after surgery have shown improvements in safety and efficiency. However, adverse drug interactions, pro-thrombotic events, poor endothelialisation, hypersensitivity and bleeding complications still occur frequently [24]. Therefore, research on a natural, non-allergic, bio- and haemo-compatible medical coating is required. In this study, we genetically designed the fusion of HSA to CD39 in order to engineer a recombinant multifunctional fusion protein which provides an ideal coating strategy for blood-contacting material. The HSA component allows adherence of our fusion protein to be passively adsorbed onto the materials, whilst the attached CD39 component prevents thrombotic actions from occurring. The data indicate that our HSA-CD39 fusion protein is stable in storage and is still highly functional in reducing platelet activation and adhesion for up to 8 weeks (Figure 3 and Figure 4B). HSA-CD39 also protects platelet activation and inflammation processes, which are commonly evoked by foreign materials such as stainless steel, Ti, nitinol (an alloy of nickel and Ti), polyurethane stents and PVC (Figure 5).

CD39 is a membrane-bound enzyme constitutively expressed on intact ECs. This NTPDase hydrolyses the nucleotides ATP and ADP [39,42,45]. CD39 has attracted major attention as a pharmacological agent [39,42,52,53]. Several studies have shown that the administration of CD39 decreases the risk of thrombosis and protects against myocardial infarction and stroke [54,55]. A hallmark study conducted in transgenic mice expressing CD39 demonstrated increased resistance to thrombosis when challenged by an acute ferric-chloride-induced injury to their carotid artery [56]. Furthermore, overexpression of CD39 in rat aortas diminishes the proliferation of smooth vascular cells and prevents neointimal formation after angioplasty [46]. However, direct injection of CD39 is associated with concentration-dependent bleeding complications [39,54]. To overcome this obstacle, our laboratory genetically fused CD39 to a single-chain antibody that was specific to activated platelets, resulting in a successful and bleeding-free targeted therapy in vivo [38,39,40]. We further investigated this construct in a murine model of myocardial ischemia/reperfusion injury, where we demonstrated that the activated-platelet-targeted CD39 provides significant myocardial protection and preserves heart function [40]. Furthermore, using CD39 mRNA, we showed the CD39 protein has active enzymatic properties and can hydrolyse ADP to AMP, thereby preventing platelet activation and proving the therapeutic potential of CD39 [42]. In this current study, we harness the enzymatic properties of CD39 and further utilise HSA for coating on several medically used materials. To demonstrate the anti-thrombotic effects of this fusion protein, we used two markers of platelet activation, the monoclonal antibody PAC-1 (specific for activated GPIIb/IIIa) and an antibody against P-selectin (anti-CD62P) (Figure S1). Upon platelet activation, GPIIb/IIIa changes from a low-affinity state to a high-binding-affinity state for fibrinogen/fibrin, thereby mediating platelet aggregation [57,58,59]. Being the most abundant platelet receptor, with the high density of 60,000 to 80,000 receptors per platelet, the activation of GPIIb/IIIa and its resulting aggregation is a main contributor to thrombosis [58,60]. P-selectin’s role in platelet aggregation is not as dominant, but it is seen as a sensitive marker of platelet activation [41]. Since most blood-contact medical device failures are due to thrombosis, our study has chosen to focus on platelet activation as a readout. Overall, our studies indicated HSA-CD39 fusion protein is highly functional in hydrolysing ADP, a major player in the platelet activation cascade, and at preventing platelet activation, adhesion and aggregation.

HSA has been widely used for the coating of medical products, possibly owing to its inferred safety and stability given its abundance in human serum [61,62,63,64]. Serum hypoalbuminemia has been observed during inflammatory processes and in cardiovascular events [29]. HSA is physiochemically stable and has been studied extensively in relation to clinical use for the maintenance of blood homeostasis in medical conditions [61,63]. Blood contact with foreign materials leads to the adherence of pro-thrombotic plasma proteins (e.g., fibrinogen) on the materials’ surfaces, but studies have shown that adsorbed albumin is able to passivate various materials, thereby providing an anti-thrombotic effect by minimising platelet adhesion [26,64,65]. Furthermore, HSA is known to provide an antioxidant effect, reducing complement cascade activation [62,65,66]. Clinically, HSA is used in combination therapy with various drugs and bioactive proteins, or as an encapsulation agent. [64]

HSA coated on an arterial polyester prosthesis (Dacron^®^) displayed reduction in platelet adhesion, less activation of the coagulation cascade and decreased formation of fibrinopeptide A, as an index for decreased thrombin action, highlighting the importance of structural design and surface chemistry [67,68]. Additionally, a HSA/polyethylenimine multilayer coating on plasma-treated PVC was shown to resist platelet adhesion effectively [69].

Ti is a material frequently used for orthopaedic implants and cardiovascular devices. Adsorption of HSA into Ti has been shown to prevent adhesion of other blood proteins and reduce bacterial adherence [70,71]. We harnessed these advantages of HSA, especially its passive binding capacity, as part of our fusion protein to improve the biocompatibility of medical devices. Our HSA-CD39 fusion protein provides protection against device-induced platelet activation and inflammation processes, and thus minimises bleeding risk and promotes adaptation of the surrounding tissue to the foreign material in situ. We demonstrate the maintained functionality of our generated fusion protein HSA-CD39 on Ti even after sterilisation with EO (Figure 5E). After radiation, sterilisation via EO is the most commonly used process in the medical device industry and is performed after standard protocols [72,73]. Therefore, we demonstrate a highly stable device coating already suited to clinical translation.

The application of therapeutic recombinant proteins for a safe, biocompatible interface on medical devices has attracted major interest in the biopharmaceutical industry. This includes the pursuit of a perfectly haemocompatible, biopassive surface and the progress in the application of active therapeutic compounds [74,75]. In the development of stents, nitinol combines the properties of elasticity, biocompatibility and the shape-memory effect, which makes it suitable for self-expanding stents. The native oxide layer formed on the surface prevents nickel ions from binding to Ti, resulting in a nickel-free environment and substantially reducing allergic reactions and toxicity [74,75]. To analyse our HSA-CD39 fusion protein in vitro, we used a flow diverter nitinol BlueOxide DERIVO embolisation device, which has been evaluated for the treatment of intracranial aneurysms in clinical trials (Figure 7 and Figure 8) [76].

In the area of coating strategies, antibodies against CD34 and CD133 coated onto stents have been evaluated in a rabbit model, showing reduced intimal proliferation and re-stenosis as compared to bare metal stents and gelatine-coated stents [62,63,75,76]. Using these antibodies to attract vascular-circulating endothelial progenitor cells (EPCs) leads to adhesion of a functional endothelial layer of EPCs on the stent surface after vascular injury. Murine monoclonal CD34-coated stents (GenousTM, OrbusNeich) were proven to be safe and enhance endothelialisation in various clinical trials [77,78]. Our study demonstrates good endothelialisation rates and a reduction in platelet activation (Figure 6).

Haemocompatibility analysis of our HSA-CD39 protein showed no influence on whole human blood. Using uncoated bare metal nitinol BlueOxide stents, we noted a significant reduction in platelet count, which also implies increased platelet aggregation. Our HSA-CD39-coated stents, on the other hand, showed no significant decrease in platelet count and additionally showed a reduced TAT complex, indicating minimal platelet aggregation and minimal coagulation cascade activation, respectively (Figure 7) [28].

Addressing the issue of the handling and storage of sensitive medical products, our data demonstrate preserved enzymatic activity of HSA-CD39 after drying, lyophilisation, coating, washing, sterilisation and 8 weeks of storage. We have shown that HSA-CD39 can be used as a new coating strategy across various devices and blood-contacting materials.

Our HSA-CD39 protein approach reduces platelet adhesion, activation and further inflammatory processes, therefore providing a great clinical advancement in the realm of bioprostheses by minimising the need for anti-thrombotic therapy, which is inherently linked to potential bleeding complications. There are some limitations to our study. We have shown that HSA-CD39 coating on our materials was present after washing steps were conducted and remained highly functional in its activity to hydrolyse ADP. However, we have not directly measured the amount of protein lost. Different materials may require other coating methods, which may expose our fusion protein to heat or other storage conditions. Although we have demonstrated that our fusion protein is more effective in reducing platelet activation, adhesion and aggregation, we have not systematically defined which of the individual components, HSA or CD39, are the cause of the described benefits. Further characterisations of HSA-CD39 should include the contributions of the individual fusion protein components. We have conducted ex vivo blood circulation and demonstrated that HSA-CD39 coating resulted in less platelet aggregation; however, future in vivo experiments will be conducted to determine the anti-thrombotic and anti-inflammatory properties of the materials post implantation. Additionally, the contribution of reduced ADP levels, in comparison to the generation of adenosine via the use of P2Y receptor inhibitors or A2A adenosine receptor blockers in vivo, will allow us to define the effects of HSA-CD39 more thoroughly. In addition, future investigations into the coating strategies, temperature changes and storage conditions, as well as a diverse range of biomaterials, will be explored.

## 5. Conclusions

In this study, we have generated a recombinant fusion protein combining the anti-thrombotic and anti-inflammatory properties of CD39 with HSA as a suitable coating for medical devices in order to reduce foreign-material-associated complications. Our newly designed HSA-CD39 fusion protein is highly functional in preventing platelet activation, adhesion and aggregation. It is also stable after EO sterilisation and can be coated onto several materials typically used in medical devices. HSA-CD39 coating can mitigate the healing process, improve the incorporation of foreign material into the surrounding tissue and reduce interactions with blood components such as coagulation proteins, platelets and leukocytes. Overall, our HSA-CD39 fusion protein is a natural bioactive interface which is highly potent in the prevention of platelet activation and inflammation; therefore, its use for medical device coating provides potential benefits for patients.

## Data Availability

Data is contained within the article or Supplementary Material.

## Supplementary Materials

The following are available online at https://www.mdpi.com/article/10.3390/pharmaceutics13091504/s1, Figure S1: Representative images of fluorescence histograms via flow cytometry demonstrating HSA-CD39 prevents platelet activation using two markers of platelet activation; Figure S2: Flow cytometry demonstrating HSA-CD39 can be dried in polystyrene tubes and stored at RT for up to 7 weeks.
