# Supplementary material for: A Recombinant Fusion Construct between Human Serum Albumin and NTPDase CD39 Allows Anti-Inflammatory and Anti-Thrombotic Coating of Medical Devices
Source: Pharmaceutics. 2021 Sep 18;13(9):1504. doi: 10.3390/pharmaceutics13091504 (PMC8466136; doi:10.3390/pharmaceutics13091504)
Supplement: Supplementary file 1 [file pharmaceutics-13-01504-s001.zip › pharmaceutics-1371117-supplementary.pdf]

pharmaceutics

MDPI

Article

Supplementary Materials: A Recombinant Fusion Construct between Human Serum Albumin and NTPDase CD39 Allows Anti-Inflammatory and Anti-Thrombotic Coating of Medical Devices

Meike-Kristin Abraham, Elena Jost, Jan David Hohmann, Amy Kate Searle, Viktoria Bongcaron, Yuyang Song, Hans Peter Wendel, Karlheinz Peter, Stefanie Krajewski, Xiaowei Wang

HSA-CD39 generation and production

HSA from ALB-pGEMT (Sino Biological Inc., Beijing, China) was PCR-modified to contain the ASC-1 and NOT-1 flanking restriction sites using the sense strand primer: 5′-TGACTAGGCGCGCCATGGATGCACACAAGA-3′ and the antisense strand primer: 5′-TAATATGCGGCCGCACCACCTCCACCTCCTCCTAAGCCTAAGGCAGCTT-3′.

After amplification by PCR, HSA was cloned into a pSectag2a vector (Invitrogen, Carlsbad, CA, USA) which contained CD39 as previously described and they were digested via their respective restriction enzymes. To increase production efficiency, HSA-CD39 was additionally cloned into a gWiz vector (Genlantis, San Diego, CA, USA). A 6x histidine tag (6x his-tag) was added to the construct. This allowed protein purification and its detection by Western blot. Ligation of the plasmid was performed with T4 ligase (NEB, Ipswich, MA, USA) at 4 °C overnight. The resultant plasmids were transformed into chemically competent *E. coli* (NEB, Ipswich, MA, USA). The transformed bacteria were grown in LB medium containing either 100 µg/ml ampicillin (pSectag2a vector) or 100 µg/ml kanamycin (gWiz vector) at 37 °C. The plasmids were purified using a Plasmid Mini Kit (Qiagen, Melbourne, Australia). A control PCR using gWiz forward primer and NOT1 reverse primer revealed correct insertion of the construct. The potential colonies obtained were confirmed by DNA sequencing. Larger DNA preparations for protein production were performed using a Plasmid Maxi Kit (Promega, Fitchburg, WI, USA).

Expression in mammalians cells and purification of HSA-CD39

Protein production via mammalian cells was performed using human kidney cells (HEK293F) in suspension cell culture in Freestyle 293 expression medium (Invitrogen, Carlsbad, CA, USA). The HEK293F cells were incubated in a shaker flask at 37 °C with 5% CO_2 at 110–140 rpm. The DNA plasmid for transfection was diluted to a ratio of 1:3 with polyethylenimine PEI (Polyscience IC, Warrington, PA, USA). 24 h prior to transfection, the HEK293F cells were diluted to a concentration of $1x10^6$ cells/ml to achieve a cell density of approximately $2x10^6$ cells/ml at the time of transfection (viability ≤95%). The ratio of Freestyle medium to the PBS transfection mixture of DNA and PEI was 9:1. 1 µg/ml DNA was added to prewarmed PBS and vortexed gently 3x. PEI was added at a concentration of 3 µg/ml and vortexed 3x. The transfection mixture was incubated for 15 min at RT and then added to the suspension culture. Glucose concentration was measured, and a final concentration of 6 g/L was obtained. This concentration was maintained during the whole of production. The culture was also supplemented with 5 g/L Lupin Peptone (Solabia, Pantin, France) on day 1 and day 5 of production. On days 3, 5 and 7, the culture was supplemented with 2 mM L-glutamine (Gibco, , Carlsbad, CA, USA). Cells were harvested on day 7 when viability was 40–50%. Afterwards they were centrifuged at 6000 g for

20 min at 4 °C and supernatant was collected for protein purification. The pre-filtered proteins were purified using an NI-NTA, a nickel-based metal affinity chromatography column (Invitrogen, , Carlsbad, CA, USA) and fast protein liquid chromatography (FPLC; BioRad, Germany) according to the manufacturer's instructions. Fractions of 1 ml were collected and dialyzed against PBS to remove immidazol.

ADP Bioluminescence assay

HSA-CD39 was incubated with 20 µM ADP for 20 min. The remaining ADP was then converted to ATP by the pyruvate kinase reaction using 40 U/ml pyruvate kinase (Sigma-Aldrich, St. Louis, MO, USA), 4 µM phosphoenolpyruvate (Sigma-Aldrich, St. Louis, MO, USA), 10 mM KCl (Sigma-Aldrich, St. Louis, MO, USA), and 40 mM MgSO4 (Sigma-Aldrich, St. Louis, MO, USA) in 40 mM tricine buffer (pH 7.75). ATP (representing the non-hydrolyzed remaining ADP) was determined in a bioluminescence assay using a microplate luminometer (Mithras LB 940, Berthold Technologies, Bad Wildbad, Germany) using the ATP bioluminescence assay kit CLS II; Roche, Basel, Switzerland) according to the manufacturer's instruction. The hydrolyzed ADP levels were determined by subtracting the obtained ADP values from the 100 µM ADP starting concentration. Standard samples containing different concentrations of ADP were also measured to establish a standard curve for the ADP levels. PBS and HSA (Alburex Human albumin 5%, CSL Behring, Hattersheim am Main, Germany) were also used as controls.

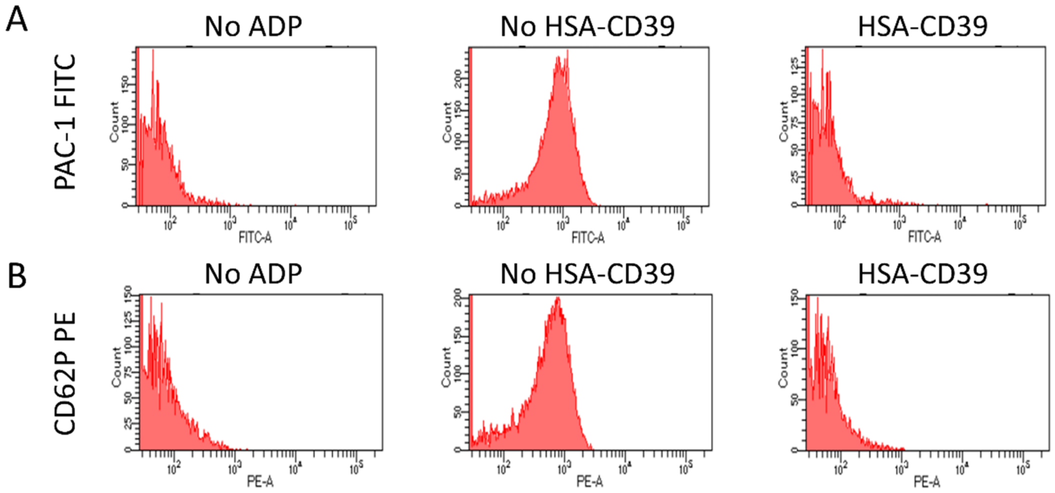

Figure S1: Representative images of fluorescence histograms via flow cytometry demonstrating HSA–CD39 prevents platelet activation using two markers of platelet activation: (A) PAC-1, a monoclonal antibody only binding to the activated conformation of the GPIIb/IIIa receptor and (B) an anti-CD62P antibody detecting the expression of P-Selectin on the activated platelet surface.

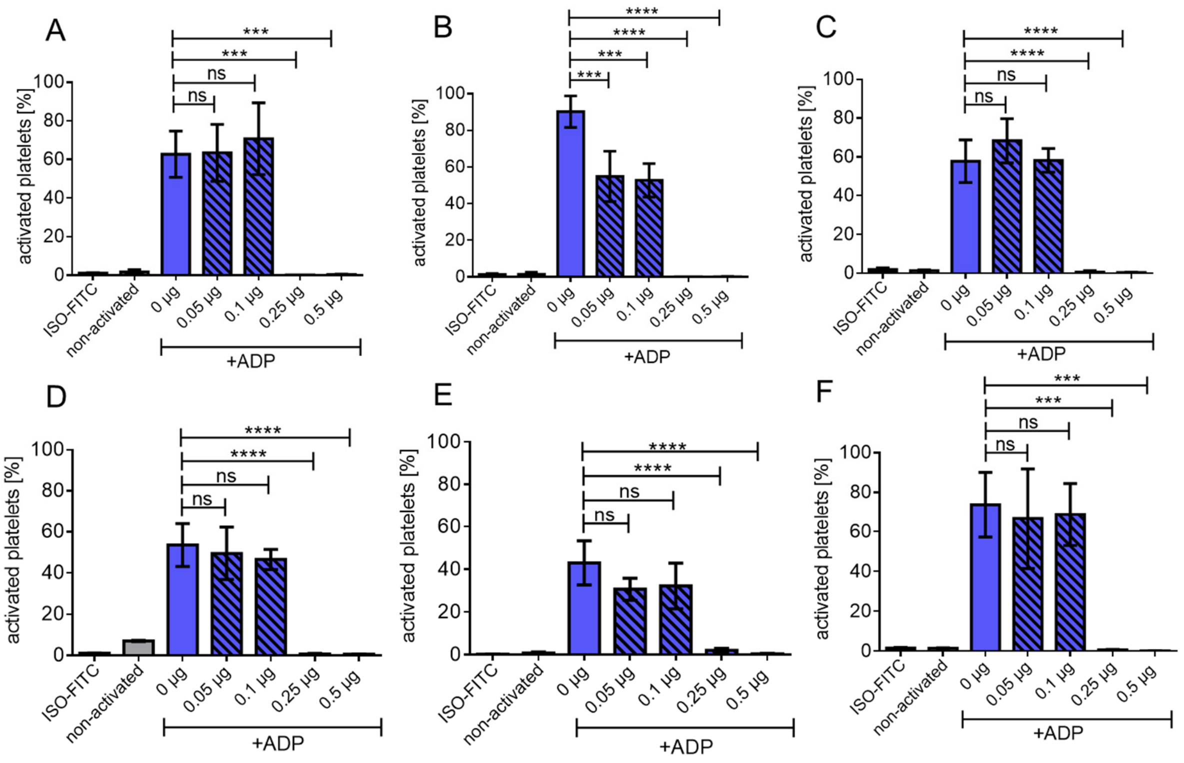

Figure S2: Flow cytometry demonstrating HSA–CD39 can be dried in polystyrene tubes and stored at RT for up to 7 weeks. HSA-CD39 retained the function to hydrolyze ADP after drying in polystyrene tubes and storage at RT. Functionality assays performed every week for 7 weeks showed good functionality post: (A) 2 weeks, (B) 3 weeks, (C) 4 weeks, (D) 5 weeks, (E) 6 weeks, (F) 7 weeks. The different groups were compared using repeated ANOVA measurements and Bonferroni post- hoc tests. Values of at least 3 independent experiments (% activated platelets ± SD, ***p<0.001 ****p<0.0001)
